# Usability of a Personal Air Pollution Monitor: Design-Feedback Iterative Cycle Study

**DOI:** 10.2196/12023

**Published:** 2018-12-21

**Authors:** Glen E Duncan, Edmund Seto, Ally R Avery, Mike Oie, Graeme Carvlin, Elena Austin, Jeffry H Shirai, Jiayang He, Byron Ockerman, Igor Novosselov

**Affiliations:** 1 Washington State University Spokane, WA United States; 2 University of Washington Seattle, WA United States; 3 Washington State University Everett, WA United States; 4 Washington State University Seattle, WA United States

**Keywords:** air pollution, methods, particulate matter, twins

## Abstract

**Background:**

There is considerable evidence that exposure to fine particulate matter (PM2.5) air pollution is associated with a variety of adverse health outcomes. However, true exposure-outcome associations are hampered by measurement issues, including compliance and exposure misclassification.

**Objective:**

This paper describes the use of the design-feedback iterative cycle to improve the design and usability of a new portable PM2.5 monitor for use in an epidemiologic study of personal air pollution measures.

**Methods:**

In total, 10 adults carried on their person a prefabricated PM2.5 monitor for 1 week over 3 waves of the iterative cycle. At the end of each wave, they participated in a 30-minute moderated focus group and completed 2 validated questionnaires on usability and views on research. The topics addressed included positives and negatives of the monitor, charging and battery life, desired features, and changes to the monitor from each previous wave. They also completed a log to record device wear time each day. The log also provided space to record any issues that may have arisen with the device or for general comments during the week of collection.

**Results:**

The major focus group topics included device size, noise, battery and charge time, and method for carrying the device. These topics formed the basis of iterative design changes; by the final cycle, the device was reasonably smaller, quieter, held a longer charge, and was more convenient to carry. System usability scores improved systematically across each wave (median scores of 50-66 on a 100-point scale), as did median daily wear time (approximately 749-789 minutes).

**Conclusions:**

Both qualitative and quantitative measures showed an improvement in device usability over the 3 waves. This study demonstrates how the design-feedback iterative cycle can be used to improve the usability of devices manufactured for use in large epidemiologic studies on personal air pollution exposures.

## Introduction

Considerable evidence proves that exposure to fine particulate matter (PM2.5) air pollution is related to various adverse health outcomes [1-9]. Despite regulation, PM2.5 remains a serious problem in the United States, where currently 20 counties affecting over 23 million people do not meet the federal PM2.5 standards [10], and even short-term increases in the PM2.5 levels may cause tens of thousands of excess deaths per year [9,11,12].

Although progress has been made in elucidating the biological mechanisms for PM2.5-related health effects, the cost, complexity, and burden on study subjects have made it difficult to conduct personal exposure assessments to better understand effects of acute and chronic exposures, where and when exposures occur, and how individual lifestyle factors affect exposures in humans under free-living conditions. Indeed, most epidemiologic studies have not measured true personal exposures. Instead, they have relied upon measurements made at central monitoring sites as exposure “surrogates,” resulting in considerable concern within the exposure assessment community as to the impact of such exposure error on disease estimates [13]. More sophisticated geospatial models have been used to try to capture spatial variations within urban areas [14] but often, the assumption is that the modeled ambient concentration at a subject’s residential address is a reasonable estimate of personal exposure, which is also incorrect because individuals are exposed to multiple locations in the course of daily living; for example, in the few studies that have used personal exposure monitoring instruments, substantial variations were found among individuals living within the same urban area and even within the same neighborhood [13,15,16]. Moreover, individuals tend to spend close to 90% of their time in indoor environments [17], and this is often not considered in air pollution epidemiologic studies. Recent meta-analyses of the issue have concluded that characteristics of the participants and their microenvironments can greatly affect the representativeness of such proxies and that greater attention is needed to evaluate the effects of measurement error [18,19].

The purpose of this study was to use the design-feedback iterative cycle to improve the usability of a portable PM2.5 monitor. This methodological paper describes the testing and refinement of the device for use in an epidemiologic study of personal air exposure measurements and clinical and biological outcomes in a large sample of twins recruited from a community-based registry. Although this study only reports on the usability aspects of the personal air pollution monitor, members of our team have published on the performance attributes of the sensor components for measuring PM2.5 and other endotoxins [20-25].

## Methods

### Recruitment

Participants for this study were drawn from the community- based Washington State Twin Registry. We chose to use twin pairs for the design-feedback iterative cycle portion of the study because twin pairs would ultimately be recruited for the epidemiologic cohort portion of the study. Only twin pairs who did not reside at the same address were eligible for the study; this ensured differential environmental exposures. For the present iterative cycle portion of the study, only pairs within the Puget Sound (King, Snohomish, Pierce, and Kitsap counties) were recruited because in-person assessments were required. The larger epidemiologic cohort portion of the study will enroll twin pairs from a wider geographic extent.

Three different versions of the personal air pollution monitor were given to participants across 3 cycles from February 2016 to August 2017. The same subjects took part in each of the 3 cycles; this ensured systematic feedback on the changes in the device over time. After each cycle, the research team used the feedback from the focus groups to modify the design of the monitor for use in the subsequent cycle.

### Device Description

Briefly, all versions of the air pollution monitor were designed by researchers at the University of Washington for personal monitoring of PM2.5 exposures. The design requirements were to create a relatively small battery-operated wearable monitor that could provide continuous timestamped and geocoded data on PM2.5 exposures during the day (ie, at least 12 hours). Although the form factor and electronic design evolved with each version of the monitors tested in the study, generally, all monitors included an onboard real-time optical particle sensor, Global Positioning System (GPS), real-time clock, data logging to a memory card, 3-axis accelerometer for physical activity tracking, and sound sensor for noise exposure monitoring. Monitors also include a microblower and plastic cartridge assembly used to collect time-integrated sample particles throughout the period that the monitor is powered on.

### Measures and Procedures

For the first cycle of the study, the twins came in for a study visit during which they received oral and written instructions on device use and for completing a daily wear log. The wear log was used to record wear time (start and stop times) for each day of the weeklong collection period. The log also provided space to record any issues that may have arisen with the device or any open-ended comments during the week of collection. They were also provided 2 validated questionnaires to assess device usability (the System Usability Scale, SUS [26]) and general views on research (Research Attitudes Questionnaire, RAQ [27,28]). The SUS contains 10 questions addressing the ease of use of the device. RAQ is a 7-question survey that measures the participants’ attitude toward medical research. Both SUS and RAQ use a 5-point Likert scale for each question with 1 being “strongly disagree” and 5 being “strongly agree.” For scoring SUS, 1 is subtracted from each odd item response and 5 from each even-numbered response. The converted responses are added up and multiplied by 2.5, providing a range of possible values from 0 (most negative experience) to 100 (most positive experience). The RAQ scale is created by summing the 7 questions to get a score that ranges from 7 to 28.

Each twin carried on their person the air pollution monitor, an ActiGraph accelerometer (ActiGraph WGT3X-BT, Pensacola, FL) for objective measurement of physical activity, and a GPS monitor (Qstarz BT-Q100XT, Taipei, Taiwan) to place exposures within a space and time framework [29]. The physical activity and GPS data are not reported in this paper because the main purpose of wearing those devices in the iterative cycle portion of the study was to ensure that it was feasible to wear all 3 devices in the epidemiologic cohort portion of the study. Participants collected data for 1 week, at which time participants returned the devices and participated in an in-person focus group. The second and third waves proceeded in the same way; however, the study materials were sent to participants via FedEx, and the focus groups were conducted via teleconference. The focus groups lasted approximately 30 minutes and were moderated by 2 trained research staff. Topics addressed included positives and negatives of the air pollution device, device charging and battery life, desired features for subsequent versions, and pros and cons of changes to the device that occurred since the previous wave.

## Results

The first focus group was completed by 5 twin pairs, the second by 4, and the final by 3 with the same participants carried over through each group. One pair was no longer interested in participating after the first wave of collection owing to increased responsibilities at work, and 1 pair was unavailable to participate in the final wave of collection because they were preparing for a multi-week vacation. The initial group of 10 consisted of 7 women and 3 men, the second group of 7 women and 1 man, and the final group of 5 women and 1 man. In total, 80% (8/10) participants were white, 80% (8/10) were aged >50 years, 80% (8/10) had completed some college, and 60% (6/10) were from households with incomes over the Washington state median based on Census data. Four of the twin pairs were monozygotic. A script for conducting the focus group was used to facilitate discussion. Notes were taken by a member of the research team, and the focus groups were recorded and transcribed by the research coordinator.

**Figure 1:**
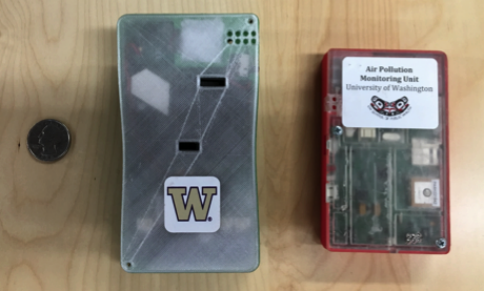
Size comparison of a personal air pollution monitor in cycle 2 (left) to cycle 3 (right) of the design feedback iterative cycle.

Text from both the focus group transcripts and the open-ended comments in the data collection log was analyzed to identify the main topics of importance. We created a corpus of both positive and negative open-ended comments and then converted the corpus to a document term matrix to determine which words were used most frequently. As shown in Table 1, the topics that emerged from the analysis across the 3 cycles of collection were device size, noise level, battery and charge time, and the method for carrying the device (belt clip, lanyard, etc). Focus group comments showed relatively slight improvements between cycle 1 and 2 with larger improvements from cycles 2 to 3; for example, the research team made the device substantially smaller from cycles 1 to 3 (initial size 5.21×0.91×2.76 inches, final size 4.25×0.88×2.56 inches; Figure 1). A muffler was added after cycle 2 to reduce the noise level of the device (from the microblower used to collect particles). In addition to the muffler, the cycle 3 device was programmed for intermittent use of the microblower for particle sampling instead of constant sampling, which also reduced the overall noise level.

The battery and charging protocol improved over the 3 cycles as well. After cycle 1, lights were added to the device to make it easier to determine when it was on and charging. The twins remarked on the helpfulness of the lights. After cycle 2, the battery was changed to extend its life and simplify the charging process. The new battery was expected to last for 16 hours, meaning the twins were able to run the device all day and then charge it overnight.

**Figure 2:**
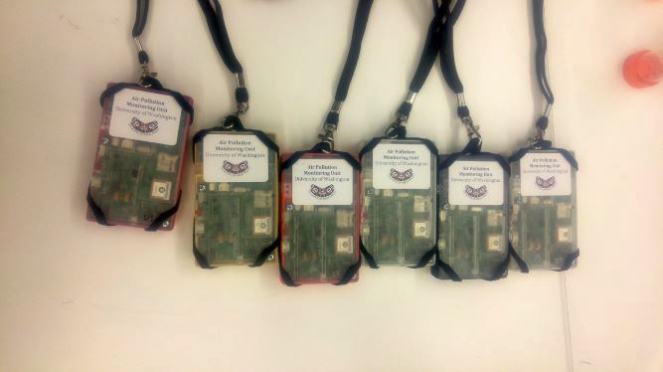
Lanyards were used in cycle 3 to allow participants to carry the air pollution monitor around the neck or attached to a bag.

A final major point of discussion was how the device was carried. In cycles 1 and 2, participants were provided with different types of belt clips. All participants had issues with the belt clips for both cycles; several participants dropped the device owing to the clips or because they could not figure out how to best attach the clips to the device. For cycle 3, the research team moved away from the belt clips and provided a lanyard for the device (Figure 2). The lanyard could be worn around the neck or attached to a bag. Reactions to the lanyard were positive, though 1 device dropped after falling out of the lanyard. The research team has already addressed this issue by securing the devices within the lanyard with a cable tie or attaching the lanyard clip through a corner strap hole on the monitor’s enclosure.

**Figure 3:**
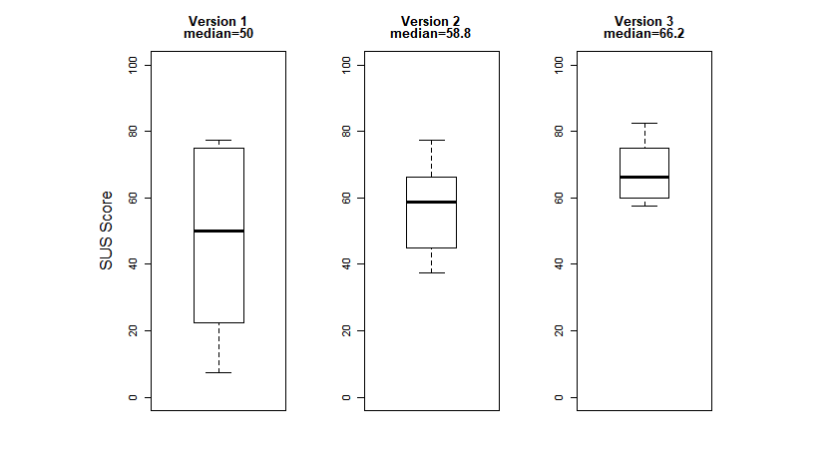
Median System Usability Scale scores (100 scale) over 3 consecutive design-feedback cycles. SUS: System Usability Scale.

**Figure 4:**
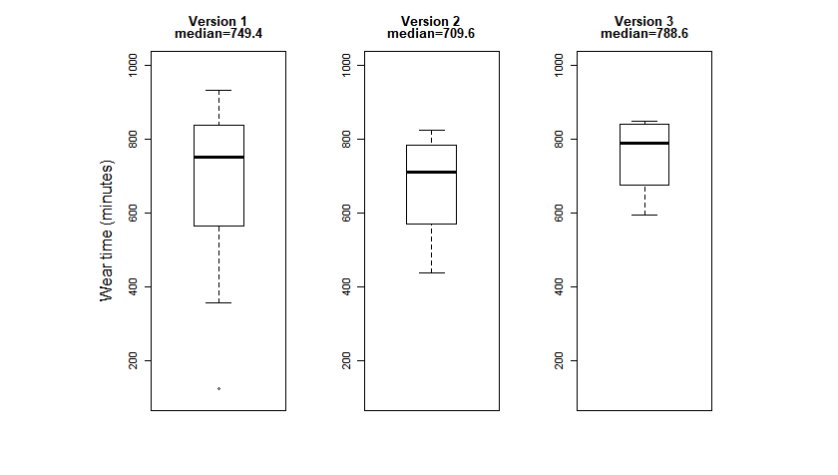
Median wear time (minutes per day) over 3 consecutive design-feedback cycles.

Figure 3 illustrates a box plot of the median wear time over the 3 cycles, showing an increase from 749 minutes per day (range 122-931 minutes) in cycle 1 to 789 minutes per day (range 594-847 minutes) in cycle 3. Figure 4 illustrates a box plot of median SUS scores over each cycle with usability scores improving from 50 in cycle 1 to 66.2 in cycle 3. In both figures, variability is shown as 1.5 times the inter-quartile range (1.5×interquartile range).

The RAQ scores demonstrated a favorable view of research in general; the average score for all participants was 24.3 (range 19-28). Moreover, focus group feedback demonstrated an interest in learning more about the purpose of the device and seeing the data being collected. Most of the participants did not feel they would want to own a device like this; however, they did bring up a few specific scenarios when the device would be helpful, such as if one had a respiratory issue or if one lived in an area with high levels of air pollution.

**Table 1:** Major thematic topics and comments from focus groups over 3 consecutive design-feedback iterative cycles for testing a personal air pollution monitor.

Major focus group topics	Cycle 1	Cycle 2	Cycle 3
Noise	Extremely irritating; concerned about others hearing it; hissing; obnoxious	Worse than first time; varied among devices; improved; hissing	Intermittent; positive reaction to cycled sampling; quieter; could still be quieter
Size	Clunky; bulky; sharp edges; cumbersome	Bulky; sharp edges	Smaller; reasonable size; liked clear device cover
Battery and charge	Couldn’t tell if device is on or off; did not stay on; unclear whether it is charged and charging	Light helpful for charging; unreliable; stopped working	Stayed charged all day; simple to charge overnight
Method for carrying	Belt clip did not work; dropped device	Better, but still not ideal; attach clip before sending	Liked the lanyard; device dropped a few times

## Discussion

### Principal Findings

This methodological paper described how the design-feedback iterative cycle was used to improve the usability of a personal air pollution monitor. In general, data gathered from the focus groups and questionnaires showed an increase in satisfaction and usability with the device over each successive iterative cycle. The device will subsequently be deployed in a large epidemiologic study enrolling 150 twin pairs to examine associations between air pollution exposures measured in time and space on markers of inflammation and cardiometabolic risk. Because twin pairs will be used in the larger epidemiologic study, we chose to use twin pairs for device testing and feedback in this study. Although it is possible that the twins may have discussed data collection with each other and thus potentially influenced compliance and survey and focus group responses, we do not believe this was a major concern. First, it is important to note that the twins did not live together, a condition of enrollment in the epidemiologic study because we are specifically interested in how “place” influences air pollution exposure. Thus, this level of intimate personal contact would not have occurred. Second, there was within-pair variability in wear time and in the comments provided on the questionnaires and during the focus groups, demonstrating that each twin member had his or her own unique experience using the device. Although twins generally agreed with one another on issues such as the size of the device and how much noise it made, these comments were made by others in the group as well. The focus group feedback and questionnaire data suggest that the device could still undergo an additional 1 or 2 iterative cycles to further improve usability, but any further changes could potentially result in a loss of functionality of the device; for example, the questionnaire data showed an increase in system usability scores over the 3 cycles. The SUS scores indicate that the user experience by the end of the design-feedback cycle had improved. Although this demonstrates a large relative improvement in usability, the final score still suggests further room for improvement. However, further changes to reduce size and noise level (still among the chief complaints) would likely have a negative effect on our ability to obtain valid and reliable data on PM2.5 air pollution. It is also important to note that participants were probably using their mobile phone or similar device as a size comparison, instead of similarly sized and commercially available personal air exposure monitors such as the MicroPEM (1.5×1.75×5 inches) or SidePak AM520 (5.1×3.7×3.1 inches).

Prior use and experience with a system or a tool can impact SUS scores on subsequent follow-up testing [30] and thus, it could be argued that participants provided better scores over time because of greater knowledge about the monitor (ie, the learning process). However, it is important to note that none of the participants had any experience with the device at baseline, so any learning that would have occurred would have been consistent across all participants. Introducing the device to novel participants at each wave would have introduced a number of important differences in subject characteristics (eg, age, sex, race or ethnicity, education level, and income level) that would have likely confounded the SUS results; therefore, we chose to keep the subjects consistent across waves. Finally, substantive changes were made to the device after each wave, including the addition of battery lights and a muffler, improved battery life, and a different carrying system for the device. All of these changes were repeatedly mentioned as positives in the focus groups and would have contributed to increased SUS scores, yet none of these changes could be attributed to increased knowledge of or experience with the device; rather, they were new additions specifically intended to improve usability.

We are not aware of any other studies that used SUS to evaluate performance in personal air pollution monitors. Thus, it is difficult to put our usability results in the context of other air pollution monitors. We identified another study that built a personal exposure monitor to measure particles as well as activity and location like ours, but it was only used for 6 hours in 1 individual [31]. Thus, once again, it is difficult to contextualize the results. The *Air Sensor Guidebook* (US Environmental Protection Agency [32]) notes that sensor performance requirements differ according to the specific application, making it difficult to compare our device to others. With respect to the increase in wear time, this is an important finding to us because our larger epidemiologic study will measure context-specific physical activity in addition to air pollution and a “valid monitoring” day in the physical activity literature is generally considered 600 minutes per day [33]. Thus, we are confident that our participants will comply with the wearing time aspect of the protocol and provide adequate physical activity data. The minimum wear time for a valid monitoring day in air pollution exposure studies is unknown, but we are confident that we will exceed the 600 minutes per day threshold, which should provide more than adequate data on personal air pollution exposures on a given day.

During the third cycle of collection, the Seattle region was experiencing an increase in smoke from wildfires in British Columbia, which was mentioned by the participants in the third focus group. They were curious as to what impact the wildfires would have on the data they were collecting. This speaks to the utility of the device; not only is the device a robust instrument for collecting PM2.5 air pollution data for research but it may also serve as a personal health monitoring tool to assess the impact of current air quality conditions in individuals with compromised health (eg, asthma). An example of the reports sent to participants is included in the Additional File, illustrating PM2.5 outdoor and personal air pollution during the data collection period for 2 members of a twin pair who are discordant for exposure. That the device may serve as a robust instrument for air pollution research is also supported by the participant’s generally favorable view of the research study with an average RAQ score of 24.3 (on a 28-point scale).

### Limitations

The main limitation of this study is the small sample size. Starting with only 5 pairs, we lost 1 pair in the second wave of the study, and 2 pairs in the third wave of the study. The study required both twins to participate and therefore, the loss of 1 member of a pair resulted in the loss of the full pair because we did not have the singleton twins collect data in the second and third waves. We also had a limited number of devices available for initial testing, which made pair-wise recruitment more difficult.

### Conclusions

We used the design-feedback iterative cycle to improve the usability of a personal air pollution monitor for subsequent deployment in an epidemiologic cohort study. As attempts are made to decrease overall regional concentrations of PM2.5, identifying hotspots of exposure both in time and space, indoors and outdoors, will become increasingly relevant to protect public health. The availability of a low-cost, validated personal monitor that can measure multiple aspects of exposure, behavior, and context may greatly enhance our future ability to study the health impacts of these policy and planning changes.

## Abbreviations

GPS: Global Positioning System
PM2.5: fine particulate matter
RAQ: Research Attitudes Questionnaire
SUS: System Usability Scale
